# COVID-19 and the expanding role of international urban search and rescue (USAR) teams: the case of the 2020 Beirut explosions

**DOI:** 10.1186/s41018-022-00116-z

**Published:** 2022-02-22

**Authors:** Yosuke Okita, Steve Glassey, Rajib Shaw

**Affiliations:** 1 Keio University, Fujisawa, Japan; 2 Public Safety Institute, Wellington, New Zealand

**Keywords:** COVID-19, International Urban Search and Rescue (USAR), INSARAG, 2020 Beirut Explosions, Beyond the Rubble

## Abstract

The paper conducts a case study on the international urban search and rescue (USAR) response to the Beirut explosions in August 2020. The incident is worth analysing because it was the first of the international USAR deployments under global travel restrictions arising from the COVID-19 pandemic. It will closely look at the activity of @fire Germany, which is self-recognised as a light USAR team, deployed to Beirut. Due to the arrangements imposed for COVID-19 prevention, it becomes more difficult for international USAR teams to arrive in affected areas quickly, meaning that the possibility for life-saving further decreases. Thus, international USAR teams must be flexible to contribute to early recovery activity after the completion of the search and rescue phase. The @fire Germany’s response gives a good example of how light international USAR teams could contribute to early recovery. Also, some countries might not want to receive or send international USAR teams due to the COVID-19 pandemic. In the COVID-19 era, strengthening national and local teams, or first responders, who are already in-country becomes critical to saving more lives.

## Introduction

On 4 August 2020, at 18:08 (local time), a warehouse containing a large amount of ammonium nitrate at the Beirut Port exploded. After the initial explosion, the subsequent explosions caused widespread damage. The explosions killed at least 200 and injured more than 6500 people (INSARAG 2020c). On the next day, the Lebanese government declared a 2-week state of emergency, and national and international urban search and rescue (USAR) teams were deployed to support the emergency efforts in the field. Widespread structural damage was reported at the port and the surrounding area. The Lebanese government estimated that the explosion had left more than 300,000 people homeless (OCHA 2020a).

Historically, international USAR teams have been mainly deployed to earthquakes and focused on search and rescue (SAR) operations extricating the victims trapped under rubble and collapsed structures. The United Nations (UN) coordination network for international USAR teams is the International Search and Rescue Advisory Group (INSARAG). The group was established in 1991 following the lessons of the 1988 Armenia earthquake where the deployed international USAR teams could not conduct their operations in a coordinated manner. The aim of INSARAG is to save more lives by developing international USAR capacity through the development of minimum team criteria, common methodology, guidelines and organising international meetings and exercises.

To achieve this objective, INSARAG from 2005 introduced its unique team classification system, INSARAG External Classification (IEC). Teams are classified as either heavy or medium level once they satisfy all the items listed in the IEC checklist. The classification levels reflect the capacity required for different building collapse environments, including light construction (light timber frame, bamboo, corrugated steel sheets, etc.), medium construction (low rise and commercial structures) and heavy construction (large, reinforced concrete and steel structures). The INSARAG methodology includes light teams, but they were not part of the IEC process as it was expected that these teams would not be internationally deployed. Thus, there was no light-level classification (IEC) by INSARAG. However, the 2015 Nepal earthquake, where many small-scale or “light” teams responded, became a catalyst to better recognise the role of light teams, and INSARAG was preparing for a light-level classification.

The deployment of international USAR teams to the Beirut explosions was not typical in that it was a deployment to a non-earthquake situation, and it was the first international USAR deployment amid the COVID-19 pandemic. Some studies have been conducted on the COVID-19 impacts on SAR operations (e.g. Koester 2020; Koester and Greatbatch 2020; Young 2020). These studies mainly covered domestic USAR operations, such as how the emergency services can provide the same level of response even during the pandemic.

International USAR operations under the COVID-19 pandemic would be further complicated. Depending on the situation, team members might be required to take the polymerase chain reaction (PCR) tests at the arrival airports. Such tests lose valuable time and may decrease the possibility of life-saving by international teams. Also, affected countries might not wish to request international USAR assistance to avoid the spread of COVID-19 brought by foreign teams, and assisting countries might not wish to send their teams to the countries where the COVID-19 cases are increasing, and their emergency service system may already be overwhelmed. Traditional international USAR deployments (e.g. large-scale teams focusing only on life-saving operations) might not always be viable in the era of COVID-19. Thus, the role of relatively small-scale teams that can also contribute to recovery tasks should be re-evaluated as an integral element of international USAR.

This study aims to discuss the expanding role of international USAR teams through a case study using the 2020 Beirut explosions where international USAR teams were deployed to a non-earthquake situation and contributed to the rescue and recovery tasks during the COVID-19 pandemic. The study closely looks at the @fire Germany team’s operation in Beirut since the team was scheduled to undergo the first IEC in light level classification in 2020, although the evaluation was postponed due to COVID-19. The study will provide some thoughts about how international USAR teams, even if they are small-scale teams, can contribute during the early recovery and capacity building of first responders, which will increase in importance in the COVID-19 era.

@fire Germany is an NGO team founded in 2002 to fight forest fires outside of Germany. They have approximately 250 registered volunteers working as firefighters and paramedics. Most of the volunteers are full-time firefighters and emergency medical services (EMS) personnel, and they get the same training as full-time firefighters or EMS staff. They also have approximately 10 trained search dogs ready for deployment. The funding for @fire deployments is mainly by donations.

## Literature review

Before the Beirut response, INSARAG has discussed the expanding role of international USAR teams. This includes international USAR teams’ contribution to recovery activity after the SAR phase finishes, called “Beyond the Rubble” and the role of “Light” international USAR teams. This section summarises the discussion among the INSARAG network so far.

### End of the SAR phase and “Beyond the Rubble”

It is generally said that the possibility of live rescue dramatically decreases after 72 h (3 days) or 96 h (4 days) after earthquakes. Thus, the decision-makers in affected countries must determine when the SAR phase should be terminated so that they can move from the rescue to the recovery phase, which often is the trigger to stand down international SAR teams, given local capacity often is able to work through less expeditiously to locate likely deceased victims. In other technical rescue disciplines such as flood rescue, decision-making processes have been developed to guide the rescue to recovery transition, such as those offered by the Department for Environment, Food and Rural Affairs (DEFRA) of the United Kingdom (UK) (DEFRA 2019).

The first INSARAG Global Meeting (IGM) held in Japan in 2010 had a session to discuss how international USAR teams can contribute to early recovery activities, utilising their resources and knowledge. As the activity is conducted beyond life-saving, it was called “Beyond the Rubble” activity among the INSARAG network (IGM 2010). The discussion about “Beyond the Rubble” continued in the subsequent INSARAG meetings. For example, the INSARAG Steering Group (ISG) Meeting in 2016 had a breakout discussion on the topic, and the DACHL [Germany (D), Austria (A), Switzerland (CH) and Luxembourg (L)] countries took the lead in drafting the concept note (ISG 2016).

The result of the discussion was reflected in the revised INSARAG guidelines in the 2020 version. In the guidelines, INSARAG defines the term “Beyond the Rubble” as the USAR teams utilising the existing capabilities to provide limited further assistance in the transition phase from SAR to recovery. As examples of the “Beyond the Rubble” tasks, contribution to coordination, assessment (e.g. structural assessment), logistics and medical assistance were listed in the guidelines. The “Beyond the Rubble” concept was added to the INSARAG guidelines, but it was also determined that “Beyond the Rubble” is a voluntary task of USAR teams (ISG 2020). Following the Christchurch earthquake, a significant contribution “Beyond the Rubble” was made by volunteer rescue teams trained in light rescue, who carried out various tasks including escorting the public back into restricted areas and reassurance to the public (Henry 2011). This supports the value of having response capacities that can contribute also to recovery activities.

### “Light” USAR teams

INSARAG has used the classification system of international USAR teams (IEC) since 2005, and teams were classified into heavy or medium teams. INSARAG guidelines have long mentioned light teams, including what training and equipment they should have, even though they were not eligible for IEC because they were supposed to be part of national or local response, any team regardless of level or classification continues to be eligible to list their team’s capability on to the INSARAG team directory through their country focal point.

When the Nepal earthquake happened in April 2015, there were 42 IEC-classified teams (only heavy and medium teams), and 76 international USAR teams deployed to Nepal. Among the 76, only 18 were IEC-classified teams (Okita and Shaw 2019), and there were many light-level teams deployed (OCHA 2015). Also, due to limited space and congestion at Kathmandu’s Tribhuvan airport, some heavy IEC-classified teams had difficulty getting landing permission (Okita and Shaw 2019). The INSARAG Team Leaders Meeting held in October 2015 discussed the Nepal earthquake response and recognised the role of light USAR teams (INSARAG Team Leaders 2015). This led to the creation of the INSARAG Light Team Working Group (LTWG) in 2016 to discuss the tasks, possible organisation and quality assurance of light teams (INSARAG LTWG 2016). The LTWG members were composed of representatives from the INSARAG regional groups, and three of them were from very experienced international light configured teams.

It is not clear whether any existing national light rescue team models have been reviewed by the LTWG. However, some members of the LTWG represent operationally experienced light teams and that experience and knowledge were reviewed for the international context. For example, in 2002, New Zealand established a national team accreditation programme for USAR Response Teams (NZRTs). The core of this programme was light USAR, although it was modelled on the Australian Category 1 (USAR Awareness) capability, with additional training in first aid, incident management and improved low height rescue operations. The programme developed a new training tier known as Category 1 Responder, which bridged the gap between the Australian Category 1 and 2 training levels (Henry 2011). The NZRT programme also replaced the legacy civil defence rescue programme that had not been updated significantly since the 1960s. Over 15 teams were established throughout the country, all comprised 15 or more volunteers and with varying capabilities beyond USAR including flood/swiftwater rescue, rope rescue, medical care, storm response and more. The programme established national competencies and a national multi-agency identification card (known as the USAR Orange Card) that provided proof of attaining such competencies. The programme created a *best practice guideline* for team accreditation which included procedural, training and equipment requirements.

In 2004, the largest national USAR exercise in New Zealand history was held in Christchurch (Exercise Pegasus04) which included heavy (Fire Service), light rescue teams (NZRTs) and international observers. A number of observations were made to improve future responses, including acknowledging the value of volunteer response (light rescue) teams being able to free up more specialised heavy rescue teams from basic tasks; the opportunity for light rescue teams to provide operational support/local knowledge to arriving international rescue teams; noting the importance of pre-event relationship building between light and heavy teams; UNDAC observers commending the diversity of capabilities beyond USAR the volunteer rescue teams brought to the exercise (Ministry of Civil Defence and Emergency Management 2004).

A Coroner’s inquest following the 2011 Christchurch earthquake also raised similar issues as those encountered in the prior exercise (Matenga 2014). This inquest was the first time in modern history where the actions of international USAR response were put under such a public spotlight, with the inquest being livestreamed around the world and numerous deficiencies being identified. The New Zealand USAR programme has made numerous improvements since, but the light rescue capacity remains sub-optimal and not integrated into the INSARAG system. A study by Henry (2011) found response teams who had been trained, equipped and accredited to national guidelines for light rescue felt frustrated that they were unable to be greater utilised in the rescue phase of the 2011 Christchurch earthquake response, this may highlight the need for enhanced recognition of light rescue or first responder capacities to ensure they are used for community benefit and to encourage local capacity building. The opportunity to use local capacity building programmes such as Community-Based Disaster Response Teams (CBDRT) has also alleviated social problems was also identified as a benefit by Cull (2019).

At the ISG Meeting in 2018, the LTWG presented the standards for light teams: 12-h operations for 5 days at one site, comprising of 17–20 members with the functions of management, search, rescue, medical and logistics (see Table 1 for the comparison of the heavy, medium and light team capability). The proposal by the LTWG was approved by the meeting, and it was also agreed that INSARAG would conduct an IEC for light teams (ISG 2018).

**Table 1:** USAR teams’ capacity level and IEC requirements. Created by the authors based on INSARAG (2020a)

Team type	Standard number of team members	Duration of operation	Number of operational sites	USAR technical capabilities	Medical capabilities
**Light**	17–20	12-h operation for 5 days	1	Dog and/or technical search; rigging and lifting	Treat team members, search dogs, and victims
**Medium**	40	24-h operation for 7 days	1	Dog and/or technical search; rigging and lifting and ability to cut structural steel	
**Heavy**	59	24-h operation for 10 days	2 sites simultaneously	Dog and technical search; rigging and lifting and ability to cut structural steel	

## Method

The study conducts a case study of the international USAR operations in the 2020 Beirut explosions, with a focus on the @fire Germany team. It reviewed the reports issued by the Office for the Coordination of Humanitarian Affairs (OCHA), European Commission (EC) and the INSARAG Information Management Working Group (IMWG) to understand the damage caused by the explosions and the response of the international teams (e.g. number of international USAR teams, coordination mechanism). Regarding the IMWG’s support during the response, the authors conducted an unstructured interview with Jeff Maunder by email (received on 17 November 2020), New Zealand USAR and the Co-chair of the IMWG, who supported the ICMS operations in Beirut remotely. As for @fire Germany’s operations, the study reviewed the mission report submitted by the team and interviewed Johannes Gust. He was deployed to Beirut as the team leader of @fire Germany and was also a member of the LTWG. The unstructured interview with him was conducted via emails (exchanged from 4 to 11 September 2020). Unless specified, the information on the overview and the response of @fire Germany is based on the report and the interview. In the interviews, the authors have asked the interviewees to freely speak about their activities during the response. The authors also interviewed Rob Davis, the team leader of the Search and Rescue Assistance in Disasters (SARAID) team who was deployed to Beirut and also the member of the LTWG, to understand the activities of the Damage Assessment Coordination Centre (DACC) and the LTWG by email (received on 23 January 2022).

## Results

### Overview of the international USAR operations in Beirut

Within 4 August, the Lebanese government requested international USAR assistance through the European Union Emergency Response Coordination Center (EU ERCC). Thirteen international USAR teams from 10 countries responded within 24 h and arrived in Beirut from 5 August, as listed in Table 2 (INSARAG 2020b). OCHA also deployed a UN Disaster Assessment and Coordination (UNDAC) team to support the Humanitarian Country Team (HCT) and the OCHA’s country office in Lebanon (INSARAG 2020c).

**Table 2:** List of international USAR teams deployed to Beirut. Created by the authors based on INSARAG (2020b). “IEC classification” means the classification level when the teams went through the IEC process and does not mean the level for the Beirut deployment. The details of each team can be found at the USAR Directory on the INSARAG website (https://www.insarag.org/directory/usar-directory/)

	Country (team name and ID)	IEC classification	Team overview in Beirut response
1	Cyprus (EMAK and MMAD)	Non-classified	10 personnel and 8 dogs
2	Czech Republic (CZERT, CZE-01)	Heavy	36 personnel and 5 dogs
3	France (FRA 02)	Heavy	56 personnel and 3 dogs
4	France (PUI, FRA-01)	Medium	20 personnel and 2 dogs
5	Germany (THW SEEBA, GER-01)	Heavy	46 personnel and 4 dogs
6	Germany (@fire, GER-10)	Non-classified	13 personnel and 2 dogs
7	Germany (I.S.A.R. Germany, GER-02)	Medium	7 personnel
8	Greece (GR/MUSAR/1ATH, GRE-10)	Non-classified	12 personnel and 1 dog
9	Netherlands (USAR.NL, NED-01)	Heavy	64 personnel and 8 dogs
10	Poland (USAR Poland, POL-01)	Heavy	43 personnel and 4 dogs
11	Qatar (QISARG, QAT-01)	Heavy	50 personnel
12	Russia (EMERCOM, RUS-01)	Heavy	80 personnel and 5 dogs
13	Turkey (AFAD Ankara, TUR-02)	Heavy	10 personnel

UNDAC teams establish an On-Site Operations Coordination Centre (OSOCC) at the centre of affected areas and support the local governments in coordinating international assistance. Before and during deployments, international USAR teams are required to update their status (e.g. monitoring, deployed, mission completed) on the Virtual OSOCC (VO) website, which is also run by OCHA to coordinate the international emergency operations. The first arriving USAR team or UNDAC team members are supposed to set up a Reception/Departure Centre (RDC) at the arrival airports, which is in charge of registering and briefing incoming international assistance teams. USAR coordination is mainly conducted at the USAR Coordination Cell (UCC), which is established at the centre of the affected area and run by UNDAC team members and international USAR teams.

In the Beirut response, the Lebanese government did not allow an RDC to be set up at the airport due to COVID-19 and because the government desired to retain full control of the crisis and felt no need for an RDC. This resulted in some teams in the country which were not known to the USAR coordination mechanism. In terms of USAR operations in the field, teams performed their standard level with extra safety measures (INSARAG 2020b).

With information management at the UCC, INSARAG has been working on establishing a more sophisticated data collection system to manage the enormous data (e.g. collapsed buildings and needs for SAR operations) quickly and allow the maximum use of the USAR team allocations. The IMWG, which was established within the INSARAG network, has developed the Information, Coordination and Management System (ICMS) that can strengthen the information management in the UCC. The ICMS was planned to be officially introduced at the beginning of 2020 (INSARAG Team Leaders 2019).

In the Beirut response, the IMWG members activated the ICMS from the early stages. It was originally expected that the trained team members within the international USAR teams run the system. Due to the COVID-19 pandemic, however, the IMWG could not run the training as expected, and there were not enough trained staff who possessed the required ICMS knowledge within international USAR teams. In addition, this was the first time where the ICMS was used in a real mission. Thus, the IMWG members supported running the ICMS remotely (INSARAG IMWG 2020). According to Jeff Maunder, the ICMS and the support provided by the IMWG members put the whole response system many hours ahead in managing the data and prioritising the assignment of the teams to have the best effect. Also, the information collected at the ICMS by the international USAR teams was also used by other entities such as the UN Environment Programme (UNEP).

The UCC was established in Beirut on 6 August 2020 in close collaboration with the Lebanese Armed Forces (LAF). The UCC was run by members from the IEC-classified teams and a liaison officer from the LAF. A member of the IMWG, who was part of the Dutch USAR team, supported the ICMS in the UCC. Recognising that UCC was a gathering place of many colleagues, the UCC staff took the necessary safety measures (e.g. facemask and social distancing, avoid shaking hands) (INSARAG 2020b). According to the summary of the team leaders’ meeting held at the UCC on 9 August, 5 days after the explosions, the Local Emergency Management Authority (LEMA) declared the end of the SAR phase. Based on this information, the UCC was closed down at 13:00 on that day.

At the coordination meeting on 9 August, it was also announced that the “Beyond the Rubble” coordination system was requested by the Lebanese authorities and that the SARAID team, the UK-based non-governmental organisation (NGO), would lead the DACC that coordinated the international structural engineers. The meeting asked if the international USAR teams could stay in Beirut and support the “Beyond the Rubble” phase. Some teams showed their willingness to support, while some other teams decided to leave the country.

### @fire Germany’s response in Beirut

#### Deployment to the Beirut explosions

As the UN General Assembly Resolution 46/182 adopted in 1991 clarifies, external assistance should be provided with the request, or at least consent, of the affected government. This is also applicable to NGO teams. The Embassy of Lebanon in Germany received the offer of assistance from @fire on the morning of 5 August 2020 the next day of the explosions. The Lebanese Ambassador in Germany, who was later designated as Prime Minister of Lebanon on 31 August 2020, accepted the offer and requested the light team of @fire. Through the Embassy of Lebanon in Germany, @fire also got information on the needs of relief materials (e.g. drugs) in the field, with a request to disseminate the information to the international SAR network. The team uploaded the information on the VO website so that the network of international disaster response officers is aware of it.

As a light USAR team, the standard composition of @fire for international deployment is to deploy 17–20 personnel. They had a modular deployment system, and the team was divided into three groups and flew to Beirut using commercial flights. There are some reasons why @fire adopts this modular system. First, they usually use commercial flights for international deployment, and thus, space and weight they can bring in are limited. Second, this arrangement allows the first group to be deployed quickly and immediately start an assessment in the field. Third, it allows them flexible deployment: decrease or increase the resources if necessary.

The first group, comprised of seven members and one search dog with approximately 600 kg of equipment, arrived in Beirut at 06:20 on 6 August 2020 by Lufthansa Airlines flight. The seven members covered management, assessment and search areas and were prepared for deployment within short notice. The second group brought six members and one search dog with approximately 600 kg of equipment, mainly for SAR purposes. They arrived at 16:25 on the same day by Middle East Airlines flight. The third group was supposed to bring heavier rescue equipment, but @fire cancelled the third group deployment because it became apparent, based on the report by the first two groups, that heavy rescue operations were unlikely. The fact that the Lebanese authorities, on 6 August, confirmed that they no longer required additional USAR teams (EC 2020) reinforces their decision. The team thus comprised 13 personnel, including one structural engineer, one medical doctor and two dogs. Teams responding below their IEC-classified level are requested to clearly state their response capacity on VO (ISG 2010). The @fire team uploaded the modified team composition on VO (although the deployment was before their IEC classification). The team participated in SAR operations at four sites and structural assessment at 30 sites. They completed the mission on 10 August (@fire 2020). See Table 3 for the chronology in the Beirut response.

**Table 3:** Chronology in the Beirut response with a focus on international USAR response (e.g. @fire, SARAID)

Date	Local time	Event/action
4 August	18:08	Explosions at the Beirut port
4 August	–	The Lebanese government requested international USAR assistance
5 August	Morning	The Embassy of Lebanon in Germany received the offer of assistance.
5 August	–	International USAR teams began to arrive in Beirut (within 24 h of the explosion).
6 August	06:20	The first group of @fire arrived.
6 August	16:25	The second group of @fire arrived.
6 August	23:00	The first group of SARAID arrived.
6 August	–	The UCC was established.
6 August	–	The Lebanese government no longer required additional SAR assistance.
8 August	09:00	Establishment of the “Beyond the Rubble” coordination system was announced.
8 August	21:00	The second group of SARAID arrived.
9 August	13:00	The LEMA declared the end of the SAR phase (5 days after the explosion), and the UCC was closed down.
10 August	–	@fire team completed their mission.
12 August	23:00	SARAID team completed their mission.

#### COVID-19

The Beirut explosions occurred amid the COVID-19 outbreak. In Lebanon, the president declared a state of emergency on 15 March 2020. On 1 July, the night-time curfew was lifted to allow businesses to open beyond midnight, but due to the increasing number of cases, the government re-imposed some preventive measures from 28 July to 10 August (OCHA 2020b). Due to the explosions, the Beirut Port was inoperable, but Beirut Rafic Hariri International Airport was open and, as of 7 August, passengers could enter the country under the conditions that:

- (Before departure from home country) passengers must have a medical certificate with a negative COVID-19 PCR test result at most 96-h before arrival.
- (After arrival in Beirut) another PCR test will be done on arrival, and passengers are subject to medical screening. Passengers must self-quarantine until the issuance of the result of the COVID-19 test (OCHA 2020b).

Due to the background of the @fire members, some members had to do a PCR test in Germany prior to departure. Upon arrival in Beirut, all team members had to do a test as requested. Regarding personal protective equipment (PPE), @fire brought 50 overalls (infection protective wear) and 1200 N-95 masks with them. When they returned to Germany, all the team members had to do a test in Frankfurt upon arrival and to separate themselves until a negative result. Depending on the background (mostly EMS personnel), some members had to do a second test in Germany. All the tests showed the results to be negative.

#### Building assessment

The team’s structural engineers conducted a field assessment of 30 buildings based on the request from the Lebanese authorities. The members applied the INSARAG building marking, and all the reports were provided to the local authorities. The INSARAG building marking is applied to the collapsed buildings to indicate search progress and avoid duplication of search efforts (Glassey 2013). This case shows that the INSARAG marking can be used in the early recovery phase as well. However, the INSARG marking was developed for SAR purposes and not for damage assessments in the recovery phase. At the INSARAG after-action review meeting after the Beirut response, it was urged that INSARAG should draft a concept paper for DACC and identify flexible standards (INSARAG 2020c). While waiting for an assignment for rescue operations, @fire also provided training for local engineers (@fire 2020).

**Fig. 1:**
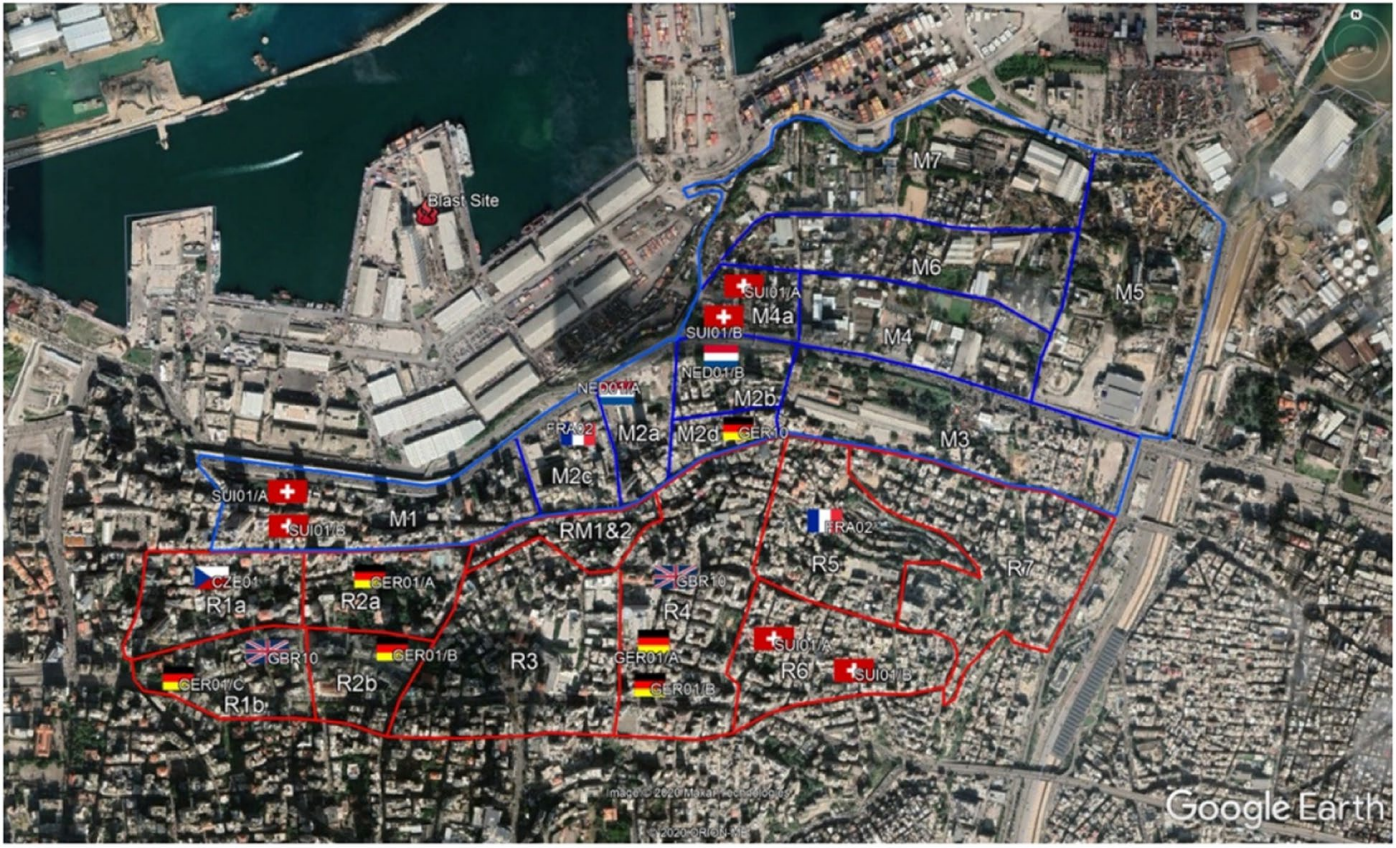
Sectors and the location of assessment teams in Beirut (provided by SARAID)

In addition, @fire provided two staff to the DACC. The role of the DACC was to support the Lebanese authorities to develop a coordination system for damage assessors and to coordinate in-country international engineers, linking them with local structural engineers (SARAID 2020). SARAID took the lead in running the DACC and coordinated the engineers from nine international teams. As a first step, they divided Beirut into 13 sectors and assigned the teams from Czech, France, Germany, Italy, the Netherlands and Switzerland (Fig. 1). The engineers assessed the buildings and reported back to SARAID. One challenge was the different methodologies used by the teams to conduct the assessment, leading to inconsistent quality. SARAID used the local assessment methodology while applying the international safety standards so that the assessment could be carried out by the people in Beirut even after the international teams had left (Findler 2020).

## Discussion

### International USAR assistance under the COVID-19 pandemic

#### Flexible deployment of international USAR teams

During the COVID-19 pandemic, there are at least four possible scenarios for international USAR deployments:

1. Affected countries do not want to request or accept foreign teams, especially from the countries with many COVID-19 cases.
2. Assisting countries hesitate to send international USAR teams to the countries with many COVID-19 cases.
3. With transit ports potentially being disrupted or other border complications, foreign teams may be reluctant to send international USAR teams in case they are unable to return home.
4. Due to COVID-19 tests before departure or after arrival, USAR teams need more time to reach the affected areas.

Affected countries have the authority to determine if they receive USAR assistance and from which countries they receive assistance (Okita and Shaw 2020b). In the Beirut response, it seems that the government prioritised USAR teams from Europe and Middle-East countries and did not decline the offers of USAR assistance from these countries that had a higher incidence of COVID-19. For the assisting side as well, it seems that the needs outweighed the risk posed by COVID-19. As for the last point, the Lebanese government requested the USAR team members to bring a negative result of a PCR test and to take another test upon arrival at the Beirut airport. For example, the @fire team members had to wait for their test results for approximately 6 to 8 h.

The possibility of live rescue dramatically decreases in the first 3 or 4 days, and “8 h” is critical. Depending on the scale of damage and building type, the needs in the field can rapidly change as time passes. In Beirut, the Lebanese government declared the end of the SAR phase after 5 days from the explosions and shifted to the “recovery” phase. Thus, teams must be prepared to contribute to “Beyond the Rubble” operations. As in the case of @fire Germany, it might be necessary to adjust the team composition and equipment based on the changing needs in the field, while INSARAG sets the standard USAR team composition by using IEC.

#### Online USAR coordination mechanism

Due to the COVID-19, it becomes more challenging to have a face-to-face coordination meeting in the affected area. INSARAG has developed the ICMS so that the data collected in the field can be quickly analysed and teams can be allocated efficiently. Although further training of staff who can run the system is needed, the system worked well in the Beirut response with the support provided by the IMWG members remotely. The online coordination mechanism works only if all the deployed USAR teams input and receive information from the system. In this sense, it is worth noting that the awareness of INSARAG and its system is increasing year by year, and the Beirut response showed much improvement in the USAR coordination from the 2015 Nepal response (INSARAG 2020b).

#### Procedures for receiving international USAR teams

It can be said that the Beirut response reminded the governments in the disaster-prone countries to revisit their procedures for receiving international USAR teams amid the COVID-19 situation. For example, the Japanese government currently (as of August 2021) request incoming international passengers from many countries to quarantine themselves for 2 weeks after they receive a negative result from their COVID-19 tests at the arrival airports. If this also applies to international USAR team members, it would be better not to request international USAR team and to prefer other types of assistance such as cash contributions. The governments in disaster-prone countries should consider whether to apply a regular procedure to international USAR teams or apply a special procedure to them.

### Strengthening first responders

As this paper indicates, some affected countries might not want to receive foreign USAR teams during the COVID-19 pandemic. Even if they receive assistance, it is predicted that international USAR teams will need more time to reach affected areas due to the COVID-19 pandemic. Thus, the role of first responders, including national and local light USAR teams, who are already in affected countries becomes more critical to save lives in the COVID-19 era.

Before the COVID-19 pandemic, it has been pointed out that international USAR teams have not saved many lives except in cases such as the 1997 Turkey earthquake and the 2010 Haiti earthquake, where more than 100 were rescued by international teams (Rom and Kelman 2020). In many earthquakes, most of the live rescues were conducted by the locals, such as relatives and neighbours (e.g. Bartolucci et al. 2019; Rom and Kelman 2020). One of the biggest reasons for this was the time to arrive in affected areas. It has been suggested that international USAR can also be used as a gesture of geo-political solidarity (Glassey 2013), despite the questionable effectiveness of such responses.

INSARAG was originally established to improve the coordination of international USAR teams in disaster rescue response, but the capacity building of national and local USAR teams has also been one of the focus areas. The 2010 INSARAG Hyogo Declaration encouraged all countries to enhance the capacity building of national USAR teams (INSARAG 2010). Based on this, INSARAG released the First Responder Training package at the 2015 ISG Meeting with support from the International Federation of Red Cross and Red Crescent Societies (IFRC) (ISG 2015). The package is available at the INSARAG website free of charge.

Though training organisations can freely use the training materials, there is no official INSARAG course certificate available to participants, and this has been seen as a deficiency in the approach. This recognition can be a strong motivation for capacity building. Hagelsteen and Becker (2019) point out that lack of motivation to change the current practices is one of the reasons behind the poor result of capacity building in disaster risk reduction (DRR). According to the guidance note of the package, in case organisations require support to deliver the course, they are suggested to contact the INSARAG’s regional group directly. However, anecdotally, despite the training package being aimed at community first responders, these volunteers in some countries struggle to find support to receive “official” training despite having country focal points. The training package refers to a “Training of Trainers” (ToT), but despite being available since 2015, anecdotal evidence suggests there has been a minimal roll-out of such courses.

The INSARAG Asia-Pacific regional group deployed USAR capacity assessment missions to Thailand in April 2015 and the Philippines in April 2016. The missions were requested by these countries as they were also interested in having IEC certification in the near future. Although both countries used the IEC checklist for the preparation for IEC and capacity building of the team for international deployment, it was not mentioned that they used the First Responder Training package for the capacity building in-country (INSARAG 2015; INSARAG 2016). It would appear the First Responder programme is not well supported within the INSARAG network; however, further research is required to substantiate this assumption.

There have been efforts by INSARAG to introduce a National Accreditation Process that allows for light teams and other capacities to be officially recognised which is encouraging. It should be noted that the INSARAG First Responder is intended for a wide range of community groups including NGOs and the emergency services, whereas the INSARAG Light Teams are specifically formed teams trained and equipped for USAR from light structures. With the involvement of the IFRC in developing the INSARAG First Responder Training package and being so active in disaster response, it is not clear why they are not part of the LTWG.

In New Zealand, recent USAR courses revealed that New Zealand Red Cross response volunteers were advised they did not need such training, despite the promotion of the INSARAG First Responder training by the IFRC (student “A”, personal communication, 15 August 2020). It would appear that the IFRC should take a stronger role in leading their own joint INSARAG/IFRC training package to its member organisations and consider becoming more active in the light rescue working group space given their focus and track record in building community-level resilience and a social licence that expects them to respond to disasters. Given the politics of heavy rescue teams often being government entities, it may be more effective to have IFRC through member societies, provide community-level ToT programmes and encourage uptake of the First Responder programme. This would likely make the training more accessible and community-focused and yield a significant increase in building local capacity.

As part of the building local capacity, Glassey and Thompson (2020) also suggest that the emergence of animal disaster response teams should be integrated into the INSARAG system by ensuring marking systems are inclusive of animal SAR (rescuing animals trapped or left behind following a disaster) and even light rescue teams that specialise in animal rescue being recognised. According to Glassey and Thompson (2020), the benefits of integrating animal rescue with human rescue is that by doing so, the additional animal rescue surge capacity acts as a force multiplier for human rescue, reduces search duplication, ensures coordinated response and minimises leaving trapped animals to cause false flags during search operations. This approach will create further community-level surge capacity if such groups are encouraged to integrate with the human rescue system and undertake the INSARAG First Responder training.

Okita and colleagues analysed the IEC processes for the Japan Disaster Relief (JDR) team, which was classified in IEC as heavy in 2010 and reclassified in 2015, and the National Search and Rescue Agency of the Republic of Indonesia (BASARNAS) team, which was classified as medium in 2019. The JDR team invited mentors from New Zealand and Australia and accepted their advice, while BASARNAS had mentors from Singapore. Both Japan and Indonesia are disaster-prone countries and are believed to already have robust USAR capability. However, they accepted the “change” brought by the international standards defined by INSARAG to be recognised internationally. As a result, it led to further strengthening of their USAR capability (e.g. the introduction of new USAR techniques). The analysis also indicated that having one IEC-classified team in the country led to strengthening national and local USAR teams in Japan and Indonesia (Okita and Shaw 2020a; Okita et al. 2018).

This study with its COVID-19 perspective supports the recommendations of Rom and Kelman (2020) and Bartolucci et al. (2019) for local capacities to be prioritised and strengthened if international USAR is to yield more cost- and lifesaving-effective responses to future disasters.

### Limitations

The study reviewed the international USAR response to the Beirut explosions, which was the first international USAR teams deployment amid the COVID-19 pandemic. Each affected country will take different responses to the offers of international USAR assistance and arrival procedures for incoming USAR teams. Thus, the findings in this study might not always apply to other countries and emergencies.

The majority of references cited in this study are from the literature produced by the UN or its member states. These references are not necessarily subject to the same scrutiny as academic peer review and our findings may be affected accordingly. On this basis, though we can report on the impact of COVID-19 on international USAR response on this occasion, our study does not provide any evidence either way as to the effectiveness of light or any other rescue responses to the Beirut explosions.

### Future research

In the COVID-19 era, the role of first responders in SAR activities will be more critical to saving lives. The study introduced some analyses where INSARAG’s classification system (IEC) got support probably because teams were motivated by being recognised by INSARAG (such as Thailand and the Philippines). Then, IEC also led to capacity building of national and local teams, or first responders, although IEC was originally developed for internationally-deployed teams (e.g. examples being the JDR team in Japan and the BASARNAS team in Indonesia). Further studies on capacity building of first responders, especially on how international USAR teams can contribute to the capacity building process, are needed.

Further research also is needed to address concerns raised by front line volunteers that despite having national focal points under the INSARAG system there can be little to no engagement, no passing on important information such as changes to marking systems, or the opportunity to be involved. The accountability of focal points needs further research to ascertain whether such anecdotes of alienation of accredited and non-accredited light rescue teams are valid. Alternatively, further research could be undertaken to ensure guidelines issued by INSARAG are being actively promoted and their implementation evaluated.

No research or reports could be found evaluating the effectiveness of the INSARAG First Responder programme, and this programme also requires further study.

## Conclusion

The study discussed the expanding role of international USAR teams and international USAR deployments during the COVID-19 pandemic, taking the example of the @fire Germany team’s response to the 2020 Beirut explosions. Due to COVID-19, international USAR teams might need more time to reach affected areas, and thus, live rescues by international USAR teams become more challenging. In the COVID-19 era, international USAR teams must be prepared so that they can contribute to “Beyond the Rubble” operations and adjust the team composition and equipment based on the changing needs in the field. The @fire’s case shows that light USAR teams can also contribute to the disaster-affected areas, utilising its mobility and flexibility.

Also, the role of the first responders becomes more important to save lives. International USAR teams need to contribute to the capacity-building process of national and local USAR teams in disaster-prone countries who are already there and immediately start rescue operations. International USAR teams, especially the IEC-classified teams who are familiar with the INSARAG methodologies, should actively support the capacity building of the first responders. The already developed materials by INSARAG, such as the INSARAG’s First Responders Training package and the IEC checklist, can be utilised for this purpose.

## Data Availability

Not applicable.

## Abbreviations

BASARNAS: Badan Nasional Pencarian dan Pertolongan (National Search and Rescue Agency of the Republic of Indonesia)
CBDRT: Community-Based Disaster Response Teams
COVID-19: Coronavirus disease caused by the SARS-CoV2 virus
DACC: Damage Assessment Coordination Centre
DACHL: Germany (D), Austria (A), Switzerland (CH) and Luxembourg (L)
DEFRA: Department for Environment, Food and Rural Affairs (United Kingdom)
DRR: Disaster risk reduction
EC: European Commission
EMS: Emergency medical services
EU ERCC: European Union Emergency Response Coordination Center
HCT: Humanitarian Country Team
ICMS: Information, Coordination and Management System
IEC: INSARAG External Classification
IFRC: International Federation of Red Cross and Red Crescent Societies
IGM: INSARAG Global Meeting
ISG: INSARAG Steering Group
IMWG: Information Management Working Group
INSARAG: International Search and Rescue Advisory Group
JDR: Japan Disaster Relief
LAF: Lebanese Armed Forces
LEMA: Local Emergency Management Authority
LTWG: Light Team Working Group
NGO: Non-governmental organisation
NZRT: New Zealand Response Team
OCHA: Office for the Coordination of Humanitarian Affairs
OSOCC: On-Site Operations Coordination Centre
PCR: Polymerase chain reaction
PPE: Personal protective equipment
RDC: Reception/Departure Centre
SAR: Search and rescue
SARAID: Search and Rescue Assistance in Disasters
ToT: Training of Trainers
UCC: USAR Coordination Cell
UK: United Kingdom
UN: United Nations
UNDAC: United Nations Disaster Assessment Coordination
UNEP: United Nations Environment Programme
USAR: Urban search and rescue
VO: Virtual OSOCC
